# Anatomic variations in basilar artery termination: a systematic review and meta-analysis with presentation of an illustrative case

**DOI:** 10.1007/s00276-026-03927-6

**Published:** 2026-07-02

**Authors:** Deepan Jayapala, Anish Narayan, Sameera Wijayawardhana, Raquel Villar-Puchades, Frederick Mariajoseph, David G. Gonsalvez, Yasith Mathangasinghe

**Affiliations:** 1 Department of Anatomy, Faculty of Medicine, University of Moratuwa, Moratuwa, Sri Lanka; 2 Centre for Human Anatomy Education, Department of Anatomy and Developmental Biology, Biomedicine Discovery Institute, Faculty of Medicine, Nursing and Health Sciences, Monash University, Clayton, Australia; 3 Department of Anatomy, Faculty of Medicine, University of Kelaniya, Kelaniya, Sri Lanka; 4 Department of Anatomy, Universitat Jaume I of Castellon, Castellon, Spain; 5 Department of Neurosurgery, Monash Health, Clayton, Australia

**Keywords:** Basilar artery, Anatomic variation, Posterior cerebral artery, Circle of willis, Terminal variations

## Abstract

**Purpose:**

Despite extensive literature on anatomical variations of the basilar artery, evidence on its termination patterns is limited. We sought out to systematically pool the prevalence of basilar artery termination variations and describe their neurointerventional implications with an illustrative case.

**Methods:**

We conducted a systematic review and meta-analysis of human basilar artery termination patterns in accordance with PRISMA guidelines. PubMed, Ovid MEDLINE, Web of Science, and Scopus were systematically searched. A risk of bias assessment was performed using the JBI critical appraisal tool. A meta-analysis of proportions was performed in R using random-effects models. Heterogeneity was assessed, and pre-specified subgroup analyses were conducted based on study type (deceased donor vs. imaging). We also present a case of basilar artery non-furcation identified during a dissection.

**Results:**

From 8469 initial records, 31 articles were eligible for quantitative analysis. The classic bifurcation pattern was most common, with a pooled prevalence of 85.94% [95% CI 56.65–96.62%]. The estimated pooled prevalences for variations were: non-furcation 8.95% [95% CI 6.20–12.74%], trifurcation 7.05% [95% CI 3.56–13.50%], quadrifurcation 5.30% [95% CI 2.16–12.45%], and pentafurcation 2.92% [95% CI 1.08–7.67%]. Hexafurcation was reported in a single study (0.87%). Imaging studies generally reported higher prevalence for variations compared to deceased donor cohorts.

**Conclusion:**

A significant proportion (14% of the population) possess variations, with non-furcation and trifurcation being most common. An understanding of these anatomical variations, as highlighted by our review and case presentation, is critical for clinical practice in neuroradiology and neurosurgery.

**Supplementary Information:**

The online version contains supplementary material available at 10.1007/s00276-026-03927-6.

## Introduction

The basilar artery (BA) represents a major supply to the cerebral posterior circulation, formed from the union of the V4 segments of the vertebral arteries and terminating through its bifurcation into the right and left posterior cerebral arteries (PCAs) [1]. The literature describes multiple variations associated with the basilar artery including variations in fenestrations, diameter, length and duplications [2–4], but inconsistently reports terminal branching patterns. Synonymous anatomy is frequently described differently such as the foetal posterior cerebral artery being referred to as an aplastic P1 or BA non-furcation.

As a result, a quantitative systematic review on terminal variations has not been conducted, with contemporary reviews prioritising the BA’s morphometric characterization such as length, diameter, and angle of formation [3]. This represents a clinically significant gap considering that a precise understanding of the vertebrobasilar system is fundamental for interpreting stroke distribution and improving diagnostic accuracy. Furthermore, the basilar artery termination point is a common site of aneurysm formation [5], making it a critical landmark in neurovascular practice.

Hence, we conducted this systematic review and meta-analysis with a dual purpose. Primarily, we aimed to provide a comprehensive quantitative synthesis of terminal basilar artery variations, calculating the pooled prevalence of each termination pattern while critically investigating how these estimates diverge based on study methodology (deceased donor dissection versus radiographic imaging). Secondarily, we sought out to synthesize a clear, step-by-step treatment framework for the most commonly reported variations by harmonising the literature on BA termination patterns.

## Methods

We conducted a systematic review and meta-analysis of proportions to estimate the prevalence of basilar artery (BA) termination patterns, adhering to the 2020 Preferred Reporting Items for Systematic Reviews and Meta-Analyses (PRISMA) guidelines [6]. The protocol was registered at the Open Science Framework (10.17605/OSF.IO/6XYS5).

### Search strategy

We searched PubMed, Ovid MEDLINE, Web of Science, and Scopus for all articles published until June 2025, using the terms “(basilar OR vertebrobasilar OR (posterior AND circulation)) AND (anatomy OR termination OR trifurcation OR quadrifurcation OR pentafurcation OR hexafurcation OR non-furcation OR variation OR variant OR “foetal PCA” OR “foetal posterior cerebral artery” OR “aplastic P1”)”. The detailed search strategy for each database is presented in Online Resource 1.

### Eligibility criteria

Inclusion criteria were studies that reported raw counts (number of events and total sample size) for at least one BA termination pattern in humans, using either donor dissection or in-vivo imaging. Exclusion criteria were: (1) reviews, editorials, opinion pieces, conference abstracts, and textbook chapters; (2) case reports (except for qualitative synthesis); (3) animal studies; (4) studies that did not explicitly describe BA termination variations; and (5) studies with data that could not be clearly extracted (e.g., unspecified foetal circulation types, failure to differentiate aplasia from hypoplasia). No language or date restrictions were applied. Three reviewers (DJ, SW, YM) independently screened titles and abstracts to identify potentially relevant studies. Full-text articles were then assessed for eligibility. Disagreements regarding eligibility were resolved through discussion among the reviewers. The authors of the original studies were contacted for further details when necessary.

### Data extraction and definitions

Three independent reviewers (DJ, SW, and YM) extracted data using a standardized collection form managed on the Covidence platform. The following details were extracted: study setting, sample size, health status of the sample population, method used (imaging vs. deceased donor), and prevalence of each termination pattern. We sought out to identify primarily non-furcation, bifurcation (normal termination), trifurcation, quadrifurcation, pentafurcation and hexafurcation. The full definitions of these is provided in Online Resource 2. 3 case control studies were included whose total sample size and summed up prevalences were extracted.

### Outcome measures

The primary outcome measures were: (1) the pooled prevalence of each termination variation and (2) the pooled prevalence of each variation stratified by study type (deceased donor vs. imaging).

### Risk of bias assessment (ROB) and GRADE certainty assessment

Risk of bias was assessed independently by two reviewers (DJ and YM) using the Joanna Briggs Institute (JBI) critical appraisal tool for prevalence data and certainty of evidence for each pooled outcome was evaluated using the GRADE framework adapted for prevalence studies.

### Statistical analysis

For each pattern, we fit random-effects models on the logit scale with Hartung–Knapp confidence intervals, then back-transformed pooled logit estimates to proportions for presentation. Heterogeneity was summarized with τ^2^, I^2^ (with 95% CIs), and Cochran’s Q. Pre-specified subgroup analyses stratified by study type were performed when ≥ 2 studies were available in a stratum. All analyses were performed in R using standard meta-analytic packages.

## Results

### Study selection and characteristics

The search yielded 8469 initial hits, from which 31 articles were deemed eligible for quantitative analysis (Online Resource 3). The majority of studies were imaging-based (n = 21, 67.74%), and only six studies (19.35%) sampled a general (apparently healthy) population. Included studies are shown in Table 1. The geographical distribution of data was broad, with the largest contributions from Europe (32.36%) and India (22.58%) (Online Resource 4).

**Table 1:** Summary of the key characteristics of the 31 studies included in the meta-analysis

Study	Title	Setting	Healthy or not healthy	Sample_type	Size	Termination variation					
						1	2	3	4	5	6
Berghout et al. [32]	Variations in intracranial arterial anatomy of the circle of Willis and their association with arteriosclerosis in patients with ischemic cerebrovascular disease	Netherlands	Not Healthy	Imaging	1126	171					
Nadeem et al. [7]	Study of Normal and Variant Anatomy of Superior Cerebellar Arteries on Cadaver Brains and Angiograms with Clinical Significance	India	Both	Both	135			11			
Alharbi et al. [35]	Anatomical study of variations in the configurations of the circle of Willis in relation to age, sex, and diameters of the components	Saudi Arabia	Not Healthy	Imaging	85	2	83				
Layegh et al. [8]	The association between vertebral artery hypoplasia and foetal-type variants of the posterior cerebral artery with imaging findings among patients with posterior circulation stroke: A single-center cross-sectional study	Iran	Not Healthy	Imaging	155	50					
Brohi et al. [33]	Radiological imaging of anatomical variations of arteries of circle of willis using magnetic resonance angiography in subset of Pakistani population	Pakistan	Healthy	Imaging	300	11					
Davidoiu et al. [9]	An Update on the Superior Cerebellar Artery Origin Type	Romania	Not Healthy	Imaging	205			42	8		
Davidoiu et al. [9]	The Foetal Type of Posterior Cerebral Artery	Romania	Not Healthy	Imaging	139	9					
Wei et al. [50]	Foetal posterior cerebral artery in pediatric and adult moyamoya disease: A single-center experience of 480 patients	China	Not Healthy	Imaging	480	23					
Kabakci et al. [40]	Anatomical Variations and Clinical Significance of the Cerebral Arterial Circle in Turkish Cadavers	Turkey	Not Specified	Deceased Donor	32	4					
Kalaiyarasi et al. [10]	Anatomical Variability in the Origin, Length and Termination of Basilar Artery and its Clinical Implications	India	Not Specified	Deceased Donor	100		93	3	3	1	
Coulier [37]	Morphologic variants of the Cerebral Arterial Circle on computed tomographic angiography (CTA): a large retrospective study	Belgium	Not Healthy	Imaging	511	49					
De Caro et al. [36]	Variants of the circle of Willis in ischemic stroke patients	Italy	Not Healthy	Imaging	1693	98					
Hsu et al. [11]	Bilateral Vertebral Artery Hypoplasia and Foetal-Type Variants of the Posterior Cerebral Artery in Acute Ischemic Stroke	China	Not Healthy	Imaging	8	4					
Shaikh et al. [47]	MRA-based evaluation of anatomical variation of circle of Willis in adult Pakistanis	Pakistan	Not Healthy	Imaging	135	19					
Nagawa et al. [12]	Terminal end Variations and Common Pathological Abnormalities of the Basilar Artery among the Ugandan Population: A Human Autopsy Study	Uganda	Not Specified	Deceased Donor	115		56	26	25	7	1
Diogo et al. [38]^*^	Low prevalence of foetal-type posterior cerebral artery in patients with basilar tip aneurysms	Portugal	Not Healthy	Imaging	396	41					
Qiu et al. [43]	MRA study on variation of the circle of willis in healthy Chinese male adults	China	Healthy	Imaging	2246	161					
Saha et al. [44]	A cadaveric study of bilateral configuration of posterior bifurcation of posterior communicating artery in Indian population	India	Not Specified	Deceased Donor	56	1					
Gunnal et al. [13]	Anatomical Variability in the Termination of the Basilar Artery in the Human Cadaveric Brain	India	Not Specified	Deceased Donor	170	5	140	9	10	6	
Krzyżewski et al. [14]	Variations and morphometric analysis of the proximal segment of the superior cerebellar artery	Poland	Healthy	Imaging	200			15	4		
Gunnal et al. [51]	Anatomical Variations of the Circulus Arteriosus in Cadaveric Human Brains	India	Not Specified	Deceased Donor	150	4		3			
Saikia et al. [45]	Study of anomalies in the circle of Willis using magnetic resonance angiography in north eastern India	India	Healthy	Imaging	70	11					
Forster et al. [39]	Anatomical Variations in the Posterior Part of the Circle of Willis and Vascular Pathology in Bilateral Thalamic Infarction	Germany	Not Healthy	Imaging	48	4					
Akgun et al. [34]	Normal anatomical features and variations of the vertebrobasilar circulation and its branches: an analysis with 64-detector row CT and 3 T MR angiographies	Turkey	Not Healthy	Imaging	135	14					
Shaban et al. [46]	Circle of Willis Variants: Foetal PCA	United States of America	Not Healthy	Imaging	536	41					
Tekale et al. [48]	Variations in the circle of willis by MR angiography	India	Healthy	Imaging	100	8					
Ogeng’o et al. [42]	Variant termination of basilar artery in a black Kenyan population	Kenya	Not Specified	Deceased Donor	173		142	18	10	3	
Veras et al. [49]	Variation of the posterior cerebral artery and its embryological explanation: a cadaveric study	Puerto Rico	Not Specified	Deceased Donor	34	2					
Hong et al. [15]^*^	Impact of posterior communicating artery on basilar artery steno-occlusive disease	South Korea	Not Healthy	Imaging	95	44					
van der Lugt et al. [41]	Accuracy of CT angiography in the assessment of a foetal origin of the posterior cerebral artery	Netherlands	Not Healthy	Imaging	51	7					
Caruso et al. [16]	Anomalies of the P1 segment of the posterior cerebral artery: early bifurcation or duplication, fenestration, common trunk with the superior cerebellar artery	France	Not Specified	Deceased Donor	100			2			

The ‘Terminal Variation’ column uses numerical codes to indicate which basilar artery termination patterns were documented in each study: (1) Non-furcation, (2) Bifurcation, (3) Trifurcation, (4) Quadrifurcation, (5) Pentafurcation, and (6) Hexafurcation. All studies are cross-sectional descriptive in nature except for * marked ones which are case control studies. All studies are published in English.

### Risk of bias and GRADE assessment

The included studies generally showed a low risk of bias, with all scoring over 55% on the JBI checklist criteria. Unclear descriptions of the health status of the sample and setting were the most common limitations (Online Resource 5). The GRADE certainty of evidence was rated Very Low for all five pooled outcomes (Online Resource 6), driven primarily by considerable statistical heterogeneity and imprecision in the rarer variation patterns.

### Meta-analysis

Across all studies, bifurcation predominated, however non-classical branching patterns constituted 14% of the cases. Funnel plots for all studies depicting heterogeneity are shown in Online Resource 7a–e.

#### Non-furcation

The overall non-furcation prevalence pooled to 8.95% (95% CI 6.20–12.74; k = 24; o = 8751; events = 783) with an I^2^ of 93.0%, indicating considerable heterogeneity (Online Resource Fig. S2a). Stratified estimates suggested a lower prevalence in deceased donor cohorts relative to imaging cohorts: deceased donor 3.62% (95% CI 1.82–7.08; k = 5; o = 442; events = 16; I^2^ = 45.9%), and imaging 10.58% (95% CI 7.22–15.26; k = 19; o = 8309; events = 767; I^2^ = 94.1%) (Online Resource 8b and c).

#### Bifurcation

For bifurcation, the overall pooled prevalence was 85.94% (95% CI 56.65–96.62%; k = 5; o = 643; events = 514; I^2^ = 94.7%) (Online Resource 9a). The deceased donor subgroup meta-analysis pooled to 79.94% (95% CI 46.99–94.71%; k = 4; o = 558; events = 431; I^2^ = 95.1%), reflecting a broad dispersion in the underlying studies (Online Resource 9b). Data from imaging cohorts were insufficient for pooling (< 2 studies).

#### Trifurcation

Trifurcation was pooled to 7.05% (95% CI 3.56–13.50; k = 9; o = 1348; events = 129; I^2^ = 87.1%) overall (Online Resource 10a). Subgroup estimates were 5.37% (95% CI 1.85–14.56; k = 6; o = 808; events = 61; I^2^ = 88.0%) in deceased donor cohorts and 12.82% (95% CI 0.08–96.55; k = 2; o = 405; events = 57; I^2^ = 92.4%) in imaging cohorts (Online Resource 10b-c).

#### Quadrifurcation

Quadrifurcation had an overall pooled prevalence of 5.30% (95% CI 2.16–12.45; k = 6; o = 963; events = 60; I^2^ = 88.7%) (Online Resource 11a). Subgroup estimates were 7.30% (95% CI 2.03–23.02%; k = 4; o = 558; events = 48; I^2^ = 89.0%) in deceased donor cohorts and 2.96% (95% CI 0.07–55.84; k = 2; o = 405; events = 12; I^2^ = 18.6%) in imaging cohorts (Online Resource 11b and c).

#### Pentafurcation

Pentafurcation pooled to 2.92% (95% CI 1.08–7.67%; k = 4; o = 558; events = 17; I^2^ = 43.9%) overall (Online Resource 12), with all data derived from deceased donor cohorts. No data were available for imaging subgroups.

#### Hexafurcation

Hexafurcation could not be meta-analyzed in any stratum due to having fewer than two eligible studies. A single study reported a prevalence of 0.87%, which is reported here narratively.

**Fig. 1:**
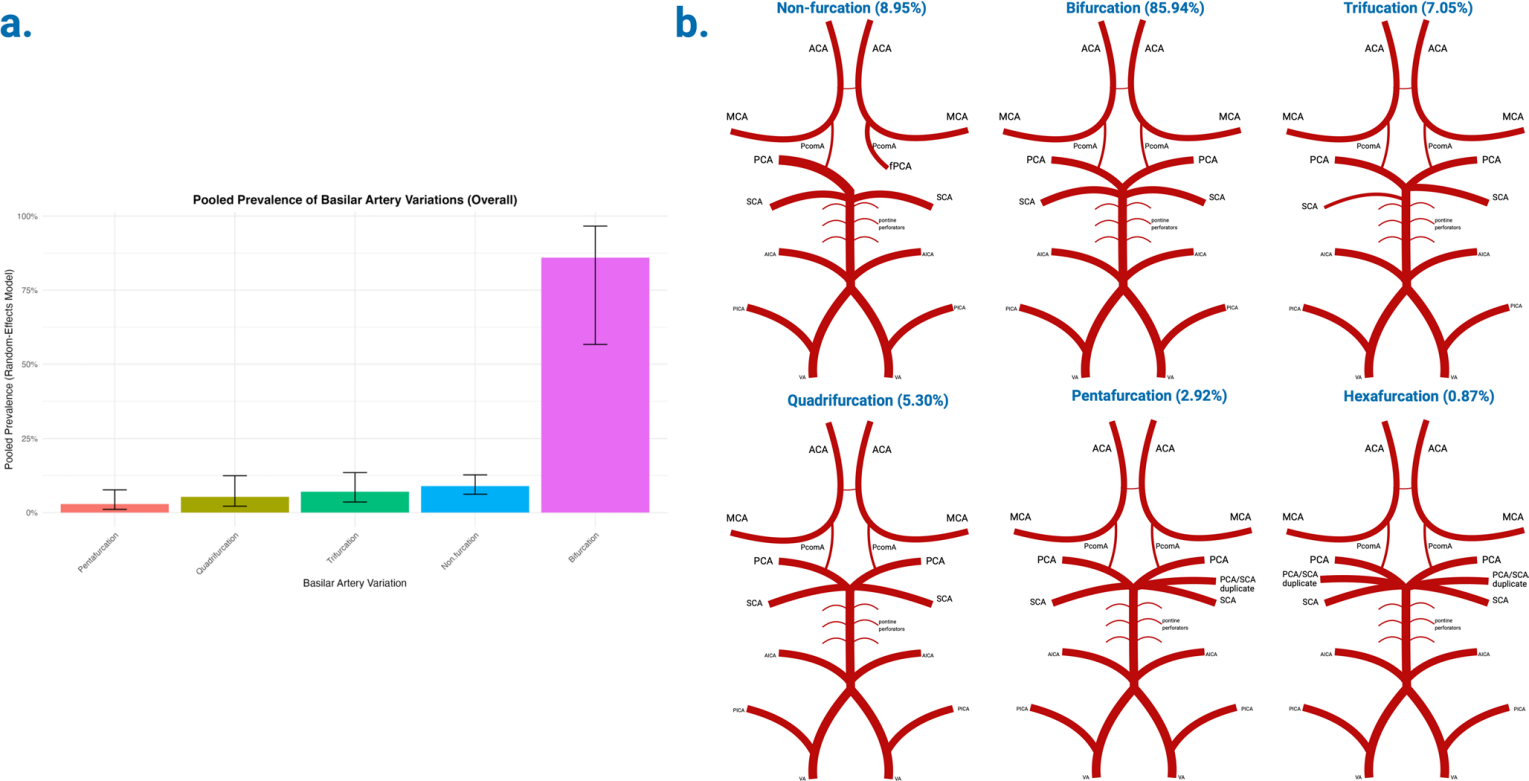
Comparative Bar Chart of Pooled Prevalences and Schematic Anatomy. **a** Random-effects pooled prevalence (95% CI) of BA termination patterns shows bifurcation as the dominant configuration, with non-furcation and trifurcation the most frequent variants among non-classical terminations. **b** Simplified schematics of the BA apex illustrate six configurations encountered in our synthesis, with pooled prevalence annotated above each panel: non-furcation (8.95%; unilateral BA termination into a single PCA), bifurcation (85.94%; classic division into right and left PCAs), trifurcation (7.05%; PCAs plus an additional trunk), quadrifurcation (5.30%), pentafurcation (2.92%), and hexafurcation (0.87%; often reflecting duplication of PCA and/or SCA trunks)

The summary bar chart (Fig. 1a) and illustration (Fig. 1b) emphasize the rank order of pooled prevalences—bifurcation >  > non-furcation ≈ trifurcation > quadrifurcation > pentafurcation—while highlighting the wide confidence intervals for the rarer patterns.

## Illustrative case presentation

**Fig. 2:**
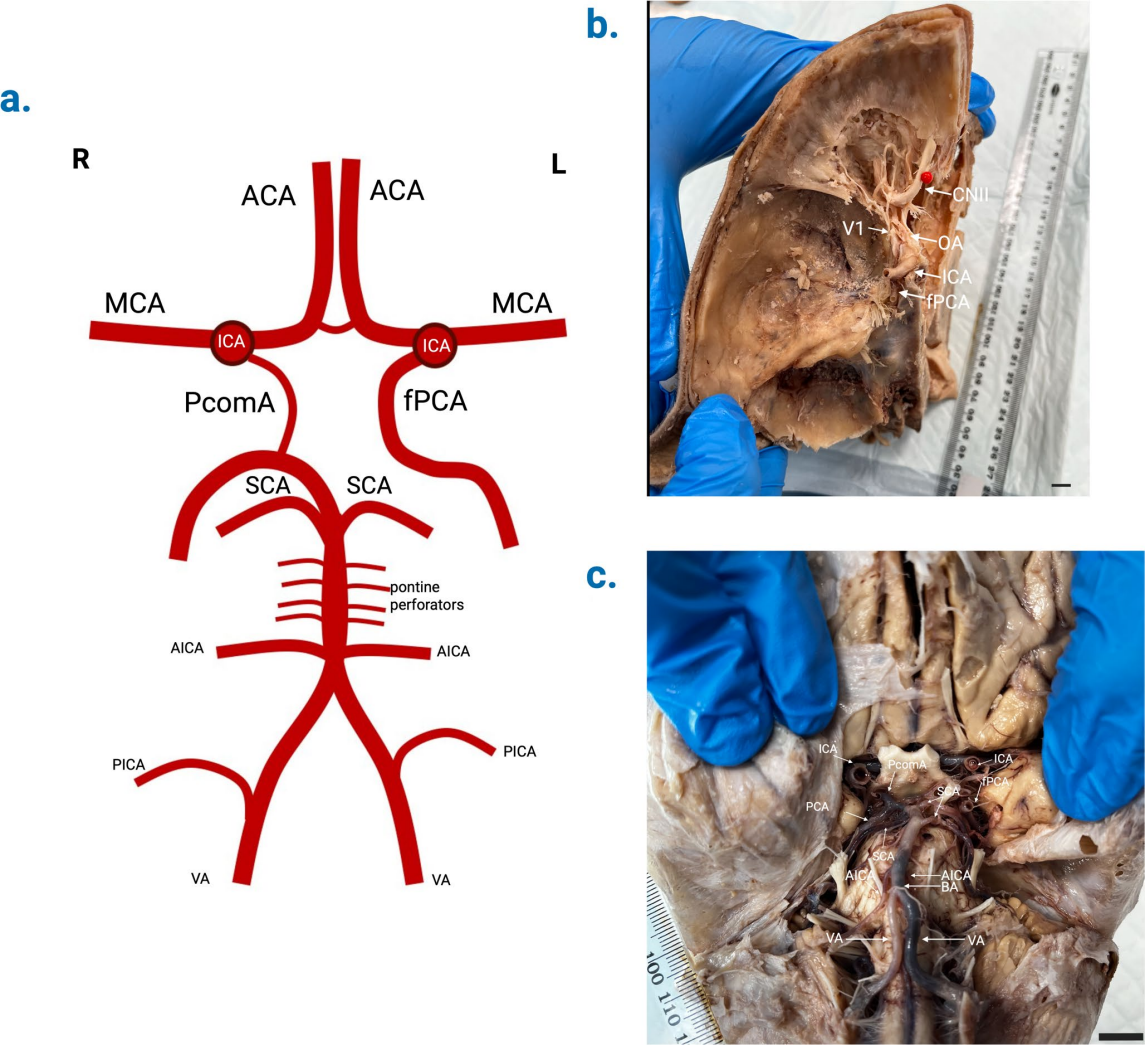
Anatomical Variation of Basilar Artery Termination from an Illustrative Case. Anatomical findings from an illustrative donor dissection, where **a** a schematic shows the basilar artery (BA) terminating unilaterally into a single right posterior cerebral artery (PCA), while the left PCA arises as a foetal-type PCA (fPCA) from the internal carotid artery (ICA). **b** A left coronal view of the half-skull shows relevant autonomic nerve structures and the origin of the fPCA from the ICA. **c** An inferior view of the brainstem shows the actual specimen with the BA non-furcation, unilateral termination, and compensatory fPCA (Scale bar = 1 cm). Abbreviations: ACA, anterior cerebral artery; AICA, anterior inferior cerebellar artery; BA, basilar artery; fPCA, foetal-type posterior cerebral artery; ICA, internal carotid artery; MCA, middle cerebral artery; PCA, posterior cerebral artery; PICA, posterior inferior cerebellar artery; PcomA, posterior communicating artery; SCA, superior cerebellar artery; VA, vertebral artery

In order to visually depict our most common variant, we present a case of fPCA presence found during a routine head and neck dissection of a female donor who died of colon cancer at the age of 72. In this dissection, the basilar artery terminated as a single PCA on the right side. The connecting P1 segment from the PCA to the basilar artery termination was absent on the left, with a prominent posterior communicating artery (PcomA) arising from the internal carotid artery (ICA), mimicking a complete foetal PCA (fPCA) variant (Fig. 2). This study was approved by the Monash University Human Research Ethics Committee (#43425).

## Discussion

### BA terminal variations

This systematic review and meta-analysis, for the first time, provides a quantitative synthesis of the variations in the termination pattern of the basilar artery. Of all the studies evaluated, bifurcation was the predominant pattern, where the basilar artery divides into a right and left posterior cerebral artery. Overall, non-furcation, where the P1 segment was absent, was the most common variation. Next was trifurcation with the most commonly observed type involving one superior cerebellar artery arising at the termination point alongside the normal bilateral two posterior cerebral arteries [7, 9, 10, 12–14]. Alternatively, the basilar artery sometimes terminated as two superior cerebellar arteries and one posterior cerebral artery; as two posterior cerebral arteries and one posterior communicating artery; or with a unilateral duplication of the posterior cerebral artery at the termination point [10, 13, 16]. Quadrifurcation was characterized by bilateral superior cerebellar arteries and bilateral posterior cerebral arteries originating at the same point, while pentafurcation included an additional superior cerebellar artery originating from the site of quadrifurcation [9, 10, 12–14]. In cases of hexafurcation, either the superior cerebellar arteries or PCAs were bilaterally duplicated [12]. These anatomical variations reflect the embryological formation of the BA and are a result of varying fusion points of its precursor paired longitudinal arteries. If the fusion occurs cranial to the origin of the SCA (superior cerebellar artery) a common bifurcation is seen. If the fusion is at the origin of the SCA, there will be a quadrifurcation. If the fusion is caudal to the origin of the SCA on one side, there will be a trifurcation, with a common trunk giving both PCA and SCA. Penta- and hexafurcation result from duplication of the SCA or PCA on one side or on both sides respectively [13].

Certain individual studies reported notably increased non-furcation prevalences of 46.3% [15], 32.3% [8], and 50% [11] compared to the overall pooled prevalence of 8.95%. Interestingly all these studies had been done on patients diagnosed with ischemic stroke which further highlights the association to vascular pathologies. Additionally, the only Ugandan study in our analysis by Nagawa et al. [12] reported significantly higher prevalences in both trifurcation (22.6%) and quadrifurcation (21.7%) compared to the pooled prevalences 7.1% and 5.3% respectively. In contrast, this study was based on deceased donors, which however reported multiple vascular pathologies including atheroma, rigidity and tortuosity.

### Prevalence discrepancies between imaging and deceased donor studies

A key finding of our analysis is the discrepancy in prevalence estimates between imaging and deceased donor studies standing as a major contributor to the high statistical heterogeneity observed. For most variations, particularly non-furcation, imaging cohorts yielded a consistently higher prevalence. This difference likely reflects inherent limitations in imaging technology, as differentiating true arterial absence (aplasia) from severe hypoplasia or an occluded vessel with minimal flow can be challenging on imaging studies [17]. Deceased donor dissection, allowing for direct visualization, likely provides a more accurate assessment of true aplasia. The health status of the study cohorts also presented a challenge, as it was often difficult to identify within deceased donor studies, limiting direct comparisons with imaging cohorts recruited for specific clinical reasons.

### Neurointerventional treatment algorithms involving the foetal posterior cerebral artery (fPCA)

#### fPCA thromboembolism diagnostic and treatment workup

Imaging workup of a potential fPCA case can be complicated on two fronts. Firstly, neurointerventionalists must differentiate between a “true” foetal PComA (where the P1 segment is aplastic) and complex duplications, such as the rare “parallel” duplication where a foetal-type branch and a conventional P1-derived PCA supply adjacent territories without communicating [18]. Advanced imaging, such as Arterial Spin Labelling (ASL) MRI, is particularly useful here; unlike conventional anatomy, fPCA territories characteristically lack physiological hyperperfusion in the medial occipital cortex and thalami, assisting identification [19].

Secondarily, due to the clinical similarity of MCA and fPCA occlusions, radiologists should not stop solely at the identification of an MCA occlusion as the possibility of an acute fPCA is still likely, signaturely indicated by a hyperdense Pcom/PCA sign [20]. Dual posterior and anterior circulation strokes are often also a clue since a true fPCA involves a shared carotid vascular tree between the PCA and MCA, meaning that an ICA thrombus or clamp threatens simultaneous anterior (MCA) and posterior (PCA) infarction [21]. Hence, these considerations and patterns can help to diagnose pathology related to or in the presence of a fPCA variant.

Treatment of fPCA thromboembolisms is similarly difficult due to the anatomical complexity of the region. Direct micro-catheterization of the fPCA is fraught with risk due to its diminutive caliber (~ 2 mm) and the high density of surrounding tuberothalamic perforators [18–25]. While modern mechanical thrombectomy is generally highly effective globally (achieving a 96% reperfusion rate) [26], the fPCA’s difficult access and tortuous anatomy can suddenly force a choice between vessel sacrifice and procedure abandonment. Hence, to systemize the approach, Ding et al. [23] present a basic algorithm for approach based on the distality of the thromboembolism. In type A thromboembolisms, the thrombus involves the opening of the fPCA and obstructs the blood flow of the entire ICA. In this configuration, the contralateral ICA compensates for the ipsilateral middle cerebral artery (MCA) and anterior cerebral artery (ACA) through the anterior communicating artery (ACOM). In type B, the thrombus involves the opening of the fPCA but does not block the blood flow of the entire ICA. The affected ICA is still able to perfuse the ipsilateral ACA and MCA. Finally, in type C, the thrombus only involves the fPCA and not the ipsilateral ICA. Patients presenting with Type A and Type B occlusions may obtain a good prognosis through endovascular treatment (EVT) which is consistent with the more approachable anatomical location. Conversely, the benefits of EVT for Type C patients are currently unclear [23]. This could indicate that proximal thromboembolisms involving fPCA are ideally treated with EVT whereas IV alteplase should be reserved for distal or tortuous cases.

#### fPCA aneurysm diagnostic and treatment workup

In perioperative planning for fPCA aneurysm treatment, several anatomical associations to the fPCA should be considered. The presence of a fPCA serves as a powerful radiological predictor for a Low-Riding Basilar Bifurcation—often situated below or within 5 mm of the clinoidal line[27]. Further, the aneurysm neck can be of varying sizes depending on if it shares the ostia of the fPCA and the ICA, or just the fPCA. Hence, the primary driver in choosing between open microsurgery and an endovascular approach is the aneurysm’s precise anatomical relationship to the fPCA origin. In cases where the pretest probability of fPCA compromise is high during endovascular intervention—most notably wide-necked aneurysms—microsurgical clipping is heavily favored over endovascular options to avoid “jailing” or occluding the fPCA origin with coils [25, 28].

For a microsurgical approach, the surgical corridor should accommodate the artery’s dominance and likely low bifurcation. Over the years, the two predominant approaches have been a minipterional (MPT) and pretemporal trans-cavernous approach. While the MPT approach is validated as a safe corridor minimizing temporal trauma [25], the pretemporal trans-cavernous approach is often superior as it offers a flatter trajectory to the interpeduncular cistern and optimizes the surgical exposure of the interpeduncular and prepontine cisterns [29] which is critical given the low bifurcation associated with fPCA anatomy. Once clipped, the patency of the fPCA can be confirmed by intraoperative sodium fluorescein angiography ensuring there is no inadvertent kinking or stenosis which can lead to occipital/thalamic infarct [25].

If coiling is selected, procedural difficulty is dictated by the “Loulida” classification [30] which uses the typological classification of type 1 involving the aneurysm ostia shared between ICA & fPCA; type 2 where the fPCA arises directly from the aneurysm dome; and type 3 where the neck originates entirely from the fPCA. Anatomically, type 2 has the highest retreatment rate (40%) potentially due to the large neck width, as it typically has the largest diameter and neck size making it the most challenging for complete occlusion. In type 1 and 2, shared or wide necks make coiling difficult due to risk of coil protrusion into the ICA or compaction, and hence stent-assisted coiling is preferred to stabilize coils in the sac and protect the parent vessel. In this case, the stent is deployed extending from the ICA distal to the fPCA origin to the ICA proximal to the fPCA origin (ICA-ICA) in most cases or in more rare cases from the ICA to the fPCA itself Then, coils are inserted into the aneurysm as much as possible to increase the packing density of the aneurysm [31]. In more complex cases, a Y-stent construct (one stent from ICA-ICA and another ICA-fPCA) can be used for durability to mechanically protect fPCA patency and prevent the jailing of perforators [28]. In the type 3 case, flow diversion using a fPCA-fPCA stenting technique is often ideal which is more logical considering the sidewall nature of the aneurysm which can be reinforced without the need to stent through a bifurcation.

### Limitations and future directions

This review has several limitations that should be acknowledged. One of the key limitations we observed was the unclear communication in the source literature regarding the absence of a termination variation, or the presence of the normal pattern, limiting the dataset available for our analysis. Another significant pitfall was the lack of a standardized nomenclature; the criteria for defining a common trunk or origin for multiple branches is an interpretation with a high risk of bias, as the visual distance for what constitutes a “common origin” may vary between observers. This subjectivity might have also led to the heterogeneity in study findings.

To address these limitations, we recommend the use of a standard nomenclature and protocol in reporting anatomical variations. For example, a terminal variation could be defined as an additional artery arising within a specified distance, such as one millimetre, of the basilar artery termination. Developing a structured observation protocol would help to achieve completeness in reporting, ensuring both the presence and absence of variations are consistently documented.

The limited number of included studies precluded the performance of sensitivity analyses, as such analyses would not give stable estimates. Similarly, while factors such as geographic region, ethnic differences, study design, differences between CTA and MRA studies, varying imaging resolutions, slice thicknesses, and methodological variabilities like the cohort selection criteria are recognized contributors to the statistical heterogeneity observed across studies, subgroup analyses examining the individual effects of these factors were not conducted, as the limited sample size would not support reliable and interpretable results. Therefore, the findings should be interpreted with caution. Furthermore, we would like to highlight that the lack of finding data is itself a result of underreporting, which we attempted to address. All these further emphasize the need for more methodologically uniform studies with larger sample sizes to establish better estimates and to expand upon the prevalence estimates reported here.

Despite the generally low risk of bias across included studies measured with the JBI criteria, the GRADE assessment rated Very Low for all five pooled outcomes. This is probably driven by the statistical heterogeneity and imprecision, with the latter being particularly due to the rarer termination variations. Consequently, with so few studies in several strata, the funnel plot interpretations and publication-bias assessments are underpowered and should be considered exploratory.

With respect to treatment, long-term angiographic follow-up studies are required, ideally comparing efficacy outcomes between treatment modalities to better standardize the ideal management algorithms depending on the specific pathological circumstance.

## Conclusion

This meta-analysis demonstrates that the classic basilar artery bifurcation, while most common, is absent in a significant proportion of the population, with approximately one in seven individuals exhibiting a variant termination pattern. This high prevalence of anatomical variation is of critical clinical importance, as it may alter stroke patterns, complicate neurosurgical and endovascular procedures, and influence cerebrovascular hemodynamics. Our analysis was limited by inconsistencies in nomenclature and incomplete reporting in the source literature, which underscores a clear need for standardization. To bridge the gap between anatomical knowledge and clinical practice, future studies should adopt uniform reporting protocols.

## Supplementary Information

Below is the link to the electronic supplementary material.

Supplementary Material 1

## Data Availability

This study is based on previously published data. All sources are cited in Table 1.
