# Correction: Anatomic variations in basilar artery termination: a systematic review and meta-analysis with presentation of an illustrative case

**DOI:** 10.1007/s00276-026-03954-3

**Published:** 2026-08-07

**Authors:** Deepan Jayapala, Anish Narayan, Sameera Wijayawardhana, Raquel Villar-Puchades, Frederick Mariajoseph, David G. Gonsalvez, Yasith Mathangasinghe

**Affiliations:** 1 Department of Anatomy, Faculty of Medicine, University of Moratuwa, Moratuwa, Sri Lanka; 2 Centre for Human Anatomy Education, Department of Anatomy and Developmental Biology, Biomedicine Discovery Institute, Faculty of Medicine, Nursing and Health Sciences, Monash University, Clayton, Australia; 3 Department of Anatomy, Faculty of Medicine, University of Kelaniya, Kelaniya, Sri Lanka; 4 Department of Anatomy, Universitat Jaume I of Castellon, Castellon, Spain; 5 Department of Neurosurgery, Monash Health, Clayton, Australia

**Correction to: Surgical and Radiologic Anatomy (2026) 48:168** 10.1007/s00276-026-03927-6

In this article, the order that the author Dr. Yasith Mathangasinghe appeared in the author list was incorrect. It has been corrected.

Correct order of author list as follows:

Deepan Jayapala^1^, Anish Narayan^2^, Sameera Wijayawardhana^3^, Raquel Villar-Puchades^4^, Frederick Mariajoseph^5^, David G Gonsalvez^2^, Yasith Mathangasinghe^2^

The Original article has been corrected.

