# Supplementary material for: Anatomic variations in basilar artery termination: a systematic review and meta-analysis with presentation of an illustrative case
Source: Surg Radiol Anat. 2026 Jul 2;48(1):168. doi: 10.1007/s00276-026-03927-6 (PMC13328238; doi:10.1007/s00276-026-03927-6)
Supplement: Supplementary file 1 — Supplementary Material 1 [file 276_2026_3927_MOESM1_ESM.docx]

**Anatomic Variations in Basilar Artery Termination: A Systematic Review and Meta-Analysis with presentation of an illustrative case**

**Authors:**Deepan Jayapala¹, Anish Narayan^2^, Sameera Wijayawardhana^3^, Raquel Villar-Puchades^4^, Frederick Mariajoseph^5^, David G Gonsalvez^2^, Yasith Mathangasinghe^2^

***Corresponding Authors***

Yasith Mathangasinghe (MBBS,PhD)

Faculty of Medicine, Monash University, Clayton, Australia

[yasith.mathangasinghe1@monash.edu](mailto:yasith.mathangasinghe1@monash.edu)

Deepan Jayapala (MBBS)

Faculty of Medicine, University of Moratuwa, Sri Lanka

[deepanj@uom.lk](mailto:deepanj@uom.lk)

Anish Narayan

Faculty of Medicine, Monash University, Clayton, Australia

[anar0024@student.monash.edu](mailto:anar0024@student.monash.edu)

+61470329556

**Supplementary Material**

**Online Resource 1. Search Strategy for Each Database.**
The detailed search terms and syntax used to query the PubMed, Ovid MEDLINE, Scopus, and Web of Science databases for the systematic review.

**Online Resource 2 -The definitions of basilar artery termination patterns**

The detailed definitions of the basilar artery termination patterns used for data extraction

**Online Resource 3. PRISMA 2020 Flow Diagram.** The study selection process for the systematic review, illustrated according to the PRISMA 2020 guidelines, detailing the number of records identified, screened, assessed for eligibility, and ultimately included in the final quantitative analysis.

**Online Resource 4. Geographical Distribution of Included Studies.**
A detailed breakdown of the geographical origin of the data from the included studies, categorized by country and region.

**Online Resource 5. Summary of Risk of Bias Assessment.**
Results of the Joanna Briggs Institute (JBI) critical appraisal tool for each included study, where each was evaluated against nine quality criteria to assess its methodological rigor and risk of bias. Responses are coded as Y (Yes), N (No), U (Unclear), or NA (Not Applicable). 1= Was the sample frame appropriate to address the target population?; 2= Were study participants sampled in an appropriate way?; 3= Was the sample size adequate?; 4= Were the study subjects and the setting described in detail?; 5= Was the data analysis conducted with sufficient coverage of the identified sample?; 6= Were valid methods used for the identification of the condition?; 7= Was the condition measured in a standard, reliable way for all participants?; 8= Was there appropriate statistical analysis?; 9= Was the response rate adequate, and if not, was the low response rate managed appropriately? Studies that did not clearly describe the health status and setting of the sample were marked as ‘Unclear’ for question 4 (Q4). Question 8 (Q8) was marked as not applicable for all studies, as direct prevalence was observed. Question 9 (Q9) was marked as not applicable for all studies involving deceased donors.

**Online Resource 6. GRADE Assessment**

Certainty ratings follow GRADE methodology adapted for prevalence studies. Starting certainty is Low for cross-sectional observational data. Risk of bias: Assessed using the JBI critical appraisal tool for prevalence studies; all included studies scored >55%. Inconsistency: Rated serious when I² >75% (considerable heterogeneity). Indirectness: All studies directly measured basilar artery termination in human subjects. Imprecision: Rated serious when the 95% CI spanned a >2-fold range or k ≤ 5 studies. Publication bias: Assessed visually via funnel plots (Supplementary Figures S7A–E).

**Online Resource 7. Funnel Plots for Assessment of Small-Study Effects.** Funnel plots of the logit-transformed prevalence from each study against its standard error, used to visually assess for heterogeneity and potential publication bias for **(a)** Non-furcation, **(b)** Bifurcation, **(c)** Trifurcation, **(d)** Quadrifurcation, and **(e)** Pentafurcation.

**Online Resource 8. Forest Plot of Non-furcation Prevalence.** Pooled prevalence for non-furcation, showing **(a)** the overall estimate (8.95%, 95% CI: 6.20–12.74%), and subgroup analyses for **(b)** deceased donors (3.62%, 95% CI: 1.82–7.08%), and **(c)** imaging-based studies (10.58%, 95% CI: 7.22–15.26%). In each plot, horizontal lines represent the 95% confidence interval for the prevalence estimate of each study, and the diamond indicates the pooled random-effects estimate.

**Online Resource 9. Forest Plot of Bifurcation Prevalence.** Pooled prevalence for the classic bifurcation pattern, showing **(a)** the overall estimate (85.94%, 95% CI: 56.65–96.62%) and **(b)** the subgroup analysis for studies on deceased donors (79.94%, 95% CI: 46.99–94.71%). In the plot, horizontal lines represent the 95% confidence interval for the prevalence estimate of each study, and the diamond indicates the pooled random-effects estimate.

**Online Resource 10. Forest Plot of Trifurcation Prevalence.** Pooled prevalence for trifurcation, showing **(a)** the overall estimate (7.05%, 95% CI: 3.56–13.50%), and subgroup analyses for **(b)** deceased donors (5.37%, 95% CI: 1.85–14.56%) and **(c)** imaging-based studies (12.82%, 95% CI: 0.08–96.55%). In the plot, horizontal lines represent the 95% confidence interval for the prevalence estimate of each study, and the diamond indicates the pooled random-effects estimate.

**Online Resource 11. Forest Plot of Quadrifurcation Prevalence.** Pooled prevalence for quadrifurcation, showing **(a)** the overall estimate (5.30%, 95% CI: 2.16–12.45%), and subgroup analyses for **(b)** deceased donors (7.30%, 95% CI: 2.03–23.02%) and **(c)** imaging-based studies (2.96%, 95% CI: 0.07–55.84%). In the plot, horizontal lines represent the 95% confidence interval for the prevalence estimate of each study, and the diamond indicates the pooled random-effects estimate.

**Online Resource 12. Forest Plot of Pentafurcation Prevalence.** Overall pooled prevalence for pentafurcation (2.92%, 95% CI: 1.08–7.67%), derived entirely from studies on deceased donors as no data were available for other subgroups. In the plot, horizontal lines represent the 95% confidence interval for the prevalence estimate of each study, and the diamond indicates the pooled random-effects estimate.

| PUBMED | (basilar[Title/Abstract] OR vertebrobasilar[Title/Abstract] OR ("posterior circulation"[Title/Abstract])) AND (anatomy[Title/Abstract] OR termination[Title/Abstract] OR trifurcation[Title/Abstract] OR quadfurcation[Title/Abstract] OR pentafurcation[Title/Abstract] OR hexafurcation[Title/Abstract] OR "non-furcation"[Title/Abstract] OR variation[Title/Abstract] OR variant[Title/Abstract] OR "fetal PCA"[Title/Abstract] OR "fetal posterior cerebral artery"[Title/Abstract]  OR "aplastic P1"[Title/Abstract]) |
| --- | --- |
| MEDLINE via Ovid | (basilar artery/ OR vertebrobasilar insufficiency/ OR (basilar OR vertebrobasilar OR (posterior ADJ circulation)).ti,ab.) AND (anatomy/ OR anatomic variation/ OR (termination OR trifurcation OR quadfurcation OR pentafurcation OR hexafurcation OR non-furcation OR variation OR variant OR "fetal PCA" OR "fetal posterior cerebral artery" OR "aplastic P1").ti,ab.) |
| Scopus | TITLE-ABS-KEY(basilar OR vertebrobasilar OR "posterior circulation")  AND TITLE-ABS-KEY(anatomy OR termination OR trifurcation OR quadfurcation OR pentafurcation OR hexafurcation OR "non-furcation" OR variation OR variant OR "fetal PCA" OR "fetal posterior cerebral artery" OR "aplastic P1") |
| Web of Science | TS=(basilar OR vertebrobasilar OR "posterior circulation")  AND TS=(anatomy OR termination OR trifurcation OR quadfurcation OR pentafurcation OR hexafurcation OR "non-furcation" OR variation OR variant OR "fetal PCA" OR "fetal posterior cerebral artery" OR "aplastic P1") |

Online Resource 1. Search Strategy for Each Database.

The detailed search terms and syntax used to query the PubMed, Ovid MEDLINE, Scopus, and Web of Science databases for the systematic review.

*.*

| **Non-furcation** | Explicit mentioning of non-furcation or unilateral P1 absence/aplasia. |
| --- | --- |
| **Bifurcation/Normal termination** | Explicit mentioning of normal bifurcation. |
| **Trifurcation** | Explicit mentioning of trifurcation, a unilateral common trunk for the SCA and PCA, or unilateral origin of the SCA from the BA termination point. |
| **Quadrifurcation** | Explicit mentioning of quadrifurcation, bilateral common trunks for the SCA and PCA, or bilateral origin of the SCAs from the BA termination point. |
| **Pentafurcation** | Explicit mentioning of pentafurcation, or a quadrifurcation pattern with unilateral duplication of either PCA or SCA, or an additional artery at the termination point. |
| **Hexafurcation** | Explicit mentioning of hexafurcation, such as bilateral duplication of the SCA and PCA originating from the termination. |

*Online Resource 2- The definitions of basilar artery termination patterns*

*The detailed definitions of the basilar artery termination patterns used for data extraction*

*Online Resource 3. PRISMA 2020 Flow Diagram.*

*The study selection process for the systematic review, illustrated according to the PRISMA 2020 guidelines, detailing the number of records identified, screened, assessed for eligibility, and ultimately included in the final quantitative analysis.*

| **Geographical territory (Included countries)** | South Asia (India, Pakistan) | Europe (France, Germany, Italy, Netherlands, Poland, Portugal, Romania, Belgium) | East Asia (China, south Korea) | Middle east ( Iran, Saudi Arabia, Turkey) | Eastern Africa (Kenya, Uganda) | America (USA, Puerto Rico) |
| --- | --- | --- | --- | --- | --- | --- |
| **Percentage of all data** | 29.03 | 32.26 | 12.90 | 12.90 | 6.45 | 6.45 |

*Online Resource 4. Geographical Distribution of Included Studies.*

*A detailed breakdown of the geographical origin of the data from the included studies, categorized by country and region.*

| Study | 1 | 2 | 3 | 4 | 5 | 6 | 7 | 8 | 9 | Cumilative |
| --- | --- | --- | --- | --- | --- | --- | --- | --- | --- | --- |
| Akgun et al., 2013 | Y | Y | Y | Y | Y | Y | Y | NA | NA | 1 |
| Alharbi et al., 2024 | Y | Y | N | Y | Y | Y | Y | NA | NA | 6/7 |
| Berghout et al.,2025 | Y | Y | Y | Y | Y | Y | Y | NA | NA | 1 |
| Caruso et al., 1991 | Y | U | N | Y | Y | Y | Y | NA | NA | 5/7 |
| Coulier, 2021 | Y | Y | Y | Y | Y | Y | Y | NA | NA | 1 |
| Davidoiu et al., 2023a | Y | Y | Y | U | Y | Y | Y | NA | NA | 6/7 |
| Davidoiu et al., 2023b | Y | Y | Y | U | Y | Y | Y | NA | NA | 6/7 |
| De Caro et al., 2021 | Y | Y | Y | Y | Y | Y | Y | NA | NA | 1 |
| Diogo et al., 2016 | Y | Y | Y | Y | Y | Y | Y | NA | NA | 1 |
| Gunnal et al., 2014 | Y | U | Y | Y | Y | Y | Y | NA | NA | 6/7 |
| Hong et al., 2009 | Y | Y | N | Y | Y | Y | Y | NA | NA | 6/7 |
| Hsu et al., 2021 | Y | Y | N | Y | Y | Y | Y | NA | NA | 6/7 |
| Krzyżewski et al., 2014 | Y | Y | Y | Y | Y | Y | Y | NA | Y | 1 |
| Qui et al., 2015 | Y | Y | Y | Y | Y | Y | Y | NA | Y | 1 |
| Saha et al., 2015 | Y | U | N | Y | Y | Y | Y | NA | NA | 5/7 |
| Shaban et al., 2013 | Y | Y | Y | Y | Y | Y | Y | NA | NA | 1 |
| Shaikh et al., 2018 | Y | Y | Y | Y | Y | Y | Y | NA | NA | 1 |
| Veras et al., 2010 | Y | U | N | U | Y | Y | Y | NA | NA | 4/7 |
| Wei et al., 2023 | Y | Y | Y | Y | Y | Y | Y | NA | NA | 1 |
| Layegh et al., 2024 | Y | Y | Y | Y | Y | Y | Y | NA | NA | 1 |
| Gunnal et al., 2014 | Y | U | Y | Y | Y | Y | Y | NA | NA | 6/7 |
| Saikia et al., 2014 | Y | Y | N | Y | Y | Y | Y | NA | U | 6/8 |
| Brohi et al., 2024 | Y | Y | Y | Y | Y | Y | Y | NA | Y | 1 |
| van der Lugt et al., 2004 | Y | Y | N | Y | Y | Y | Y | NA | NA | 6/7 |
| Forster et al., 2014 | Y | Y | N | U | Y | Y | Y | NA | NA | 5/7 |
| Nadeem et al., 2025 | Y | U | Y | Y | Y | Y | Y | NA | Y | 7/8 |
| Kabakci et al., 2023 | Y | U | N | Y | Y | Y | Y | NA | NA | 5/7 |
| Nagawa et al., 2018 | Y | U | N | Y | Y | Y | Y | NA | NA | 5/7 |
| Tekale et al., 2013 | Y | Y | N | Y | Y | Y | Y | NA | U | 6/8 |
| Ogeng'o et al., 2012 | Y | U | Y | Y | Y | Y | Y | NA | NA | 6/7 |
| Kalaiyarasi et al.,2022 | Y | U | N | Y | Y | Y | Y | NA | NA | 5/7 |

*Online Resource 5. Summary of Risk of Bias Assessment.*

*Results of the Joanna Briggs Institute (JBI) critical appraisal tool for each included study, where each was evaluated against nine quality criteria to assess its methodological rigor and risk of bias. Responses are coded as Y (Yes), N (No), U (Unclear), or NA (Not Applicable). 1= Was the sample frame appropriate to address the target population?; 2= Were study participants sampled in an appropriate way?; 3= Was the sample size adequate?; 4= Were the study subjects and the setting described in detail?; 5= Was the data analysis conducted with sufficient coverage of the identified sample?; 6= Were valid methods used for the identification of the condition?; 7= Was the condition measured in a standard, reliable way for all participants?; 8= Was there appropriate statistical analysis?; 9= Was the response rate adequate, and if not, was the low response rate managed appropriately?* *Studies that did not clearly describe the health status and setting of the sample were marked as ‘Unclear’ for question 4 (Q4). Question 8 (Q8) was marked as not applicable for all studies, as direct prevalence was observed. Question 9 (Q9) was marked as not applicable for all studies involving deceased donors.*

| **Outcome** | **k (events)** | **Risk of Bias** | **Inconsistency** | **Indirectness** | **Imprecision** | **Publication Bias** | **Pooled Prevalence (95% CI)** | **Certainty** |
| --- | --- | --- | --- | --- | --- | --- | --- | --- |
| **Non‑furcation** | 24 (783) | **Not serious**  *All studies scored >55% on JBI; low risk overall* | **Serious**  *I²=93%; considerable heterogeneity, partly explained by study type subgroup* | **Not serious**  *Studies directly measured BA termination in human subjects* | **Not serious**  *Large total sample (k=24); narrow CI relative to estimate* | **Undetected**  *Funnel plot assessed (Suppl. Fig. S7A)* | 8.95% (95% CI: 6.20–12.74) | **Very Low** ⬤○○○ |
| **Bifurcation** | 5 (514) | **Not serious**  *All studies scored >55% on JBI* | **Serious**  *I²=94.7%; very wide prediction interval* | **Not serious**  *Direct measurement of BA termination* | **Serious**  *Only k=5 studies; wide CI (56.65–96.62%)* | **Undetected**  *Funnel plot assessed (Suppl. Fig. S7B)* | 85.94% (95% CI: 56.65–96.62%) | **Very Low** ⬤○○○ |
| **Trifurcation** | 9 (129) | **Not serious**  *All studies scored >55% on JBI* | **Serious**  *I²=87.1%; substantial heterogeneity* | **Not serious**  *Direct measurement of BA termination* | **Serious**  *Moderate k=9 but wide CI (3.56–13.50%)* | **Undetected**  *Funnel plot assessed (Suppl. Fig. S7C)* | 7.05% (95% CI: 3.56–13.50) | **Very Low** ⬤○○○ |
| **Quadrifurcation** | 6 (60) | **Not serious**  *All studies scored >55% on JBI* | **Serious**  *I²=88.7%; considerable heterogeneity* | **Not serious**  *Direct measurement of BA termination* | **Serious**  *Only k=6 and wide CI (2.03–23.02%)* | **Undetected**  *Funnel plot assessed (Suppl. Fig. S7D)* | 5.30% (95% CI: 2.16–12.45) | **Very Low** ⬤○○○ |
| **Pentafurcation** | 4 (17) | **Not serious**  *All studies scored >55% on JBI* | **Not serious**  *I²=43.9%; moderate, below 50% threshold* | **Not serious**  *Direct measurement of BA termination* | **Serious**  *Only k=4 studies, all deceased donor; wide CI (1.08–7.67%)* | **Undetected**  *Funnel plot assessed (Suppl. Fig. S7E)* | 2.92% (95% CI: 1.08–7.67%) | **Very Low** ⬤○○○ |

***Online Resource 6****. Summary of GRADE Certainty assessment*

*Certainty ratings follow GRADE methodology adapted for prevalence studies. Starting certainty is Low for cross-sectional observational data. Risk of bias: Assessed using the JBI critical appraisal tool for prevalence studies; all included studies scored >55%. Inconsistency: Rated serious when I² >75% (considerable heterogeneity). Indirectness: All studies directly measured basilar artery termination in human subjects. Imprecision: Rated serious when the 95% CI spanned a >2-fold range or k ≤ 5 studies. Publication bias: Assessed visually via funnel plots (Online Resource 7a–e). Sensitivity analyses were not performed as the limited number of studies precluded meaningful assessment and heterogeneity was primarily attributable to methodological differences between study types rather than individual outlier studies*


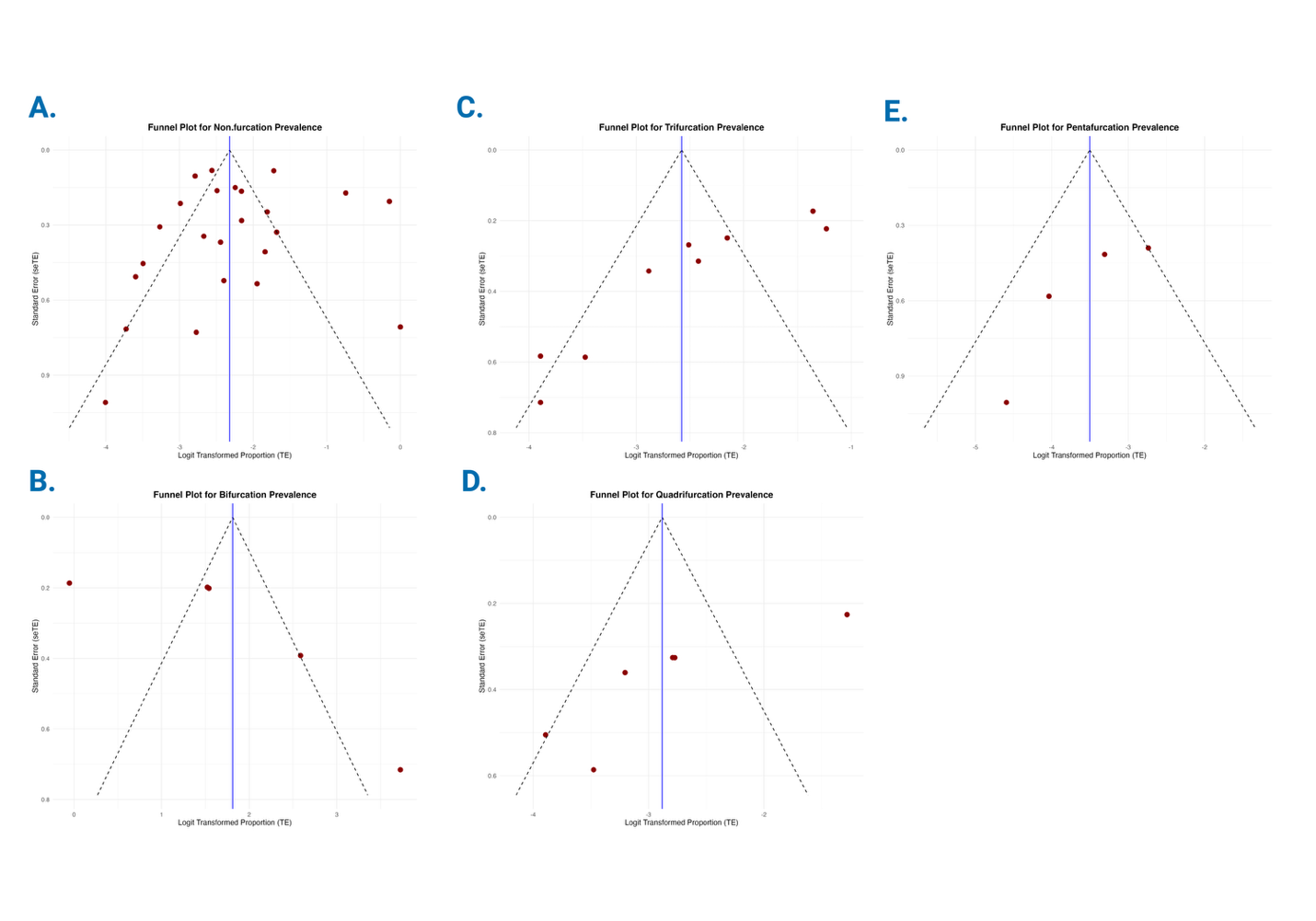


**e.**

**d.**

**c.**

**b.**

**a.**

***Online Resource 7. Funnel Plots for Assessment of Small-Study Effects.*** *Funnel plots of the logit-transformed prevalence from each study against its standard error, used to visually assess for heterogeneity and potential publication bias for****(a)****Non-furcation,****(b)****Bifurcation,****(c)****Trifurcation,****(d)****Quadrifurcation, and****(e)****Pentafurcation.*

**c.**


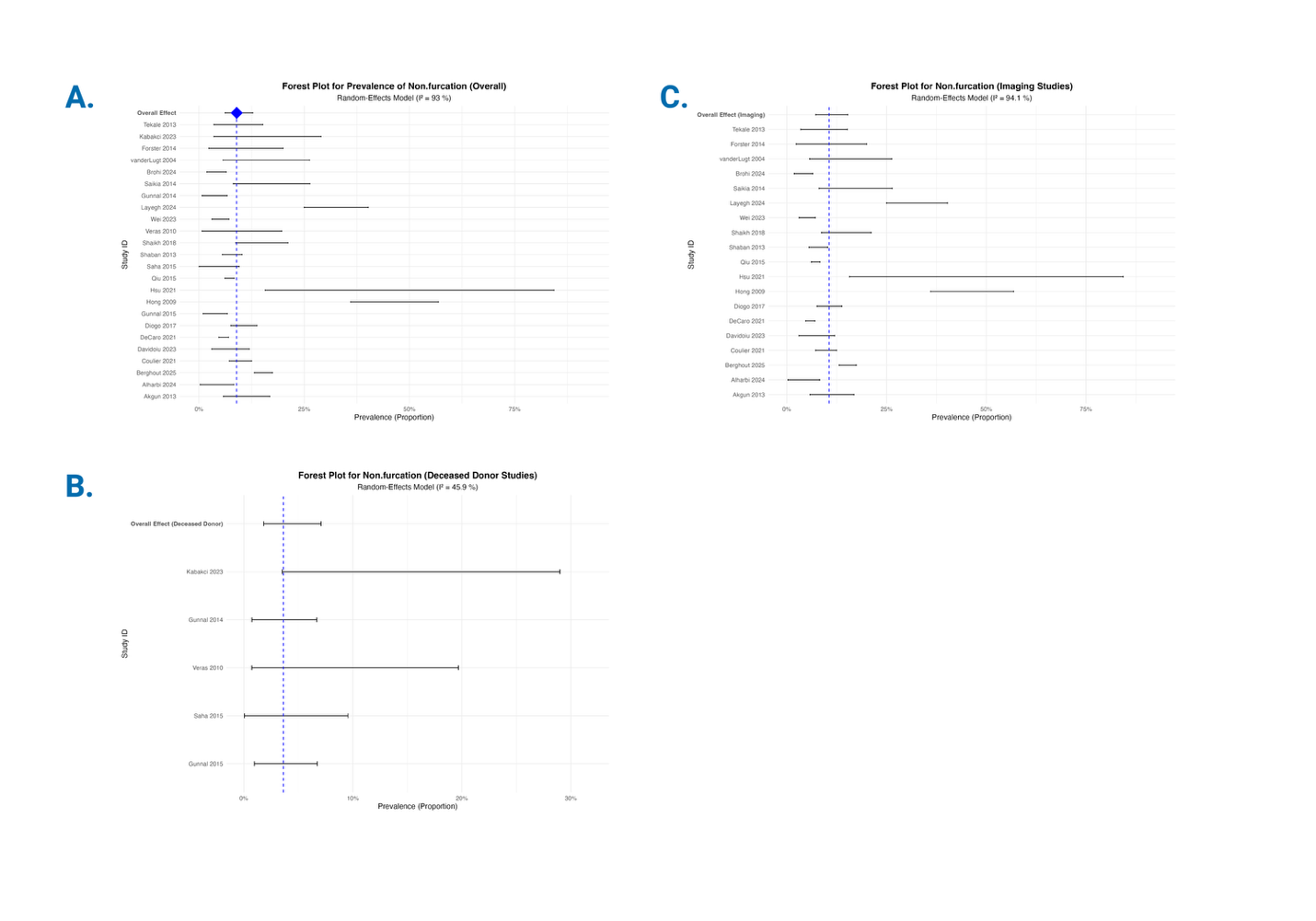


**a.**

**b.**

***Online Resource 8. Forest Plot of Non-furcation Prevalence.*** *Pooled prevalence for non-furcation, showing****(a)****the overall estimate (8.95%, 95% CI: 6.20–12.74%), and subgroup analyses for****(b)****deceased donors (3.62%, 95% CI: 1.82–7.08%), and****(c)****imaging-based studies (10.58%, 95% CI: 7.22–15.26%). In each plot, horizontal lines represent the 95% confidence interval for the prevalence estimate of each study, and the diamond indicates the pooled random-effects estimate.*


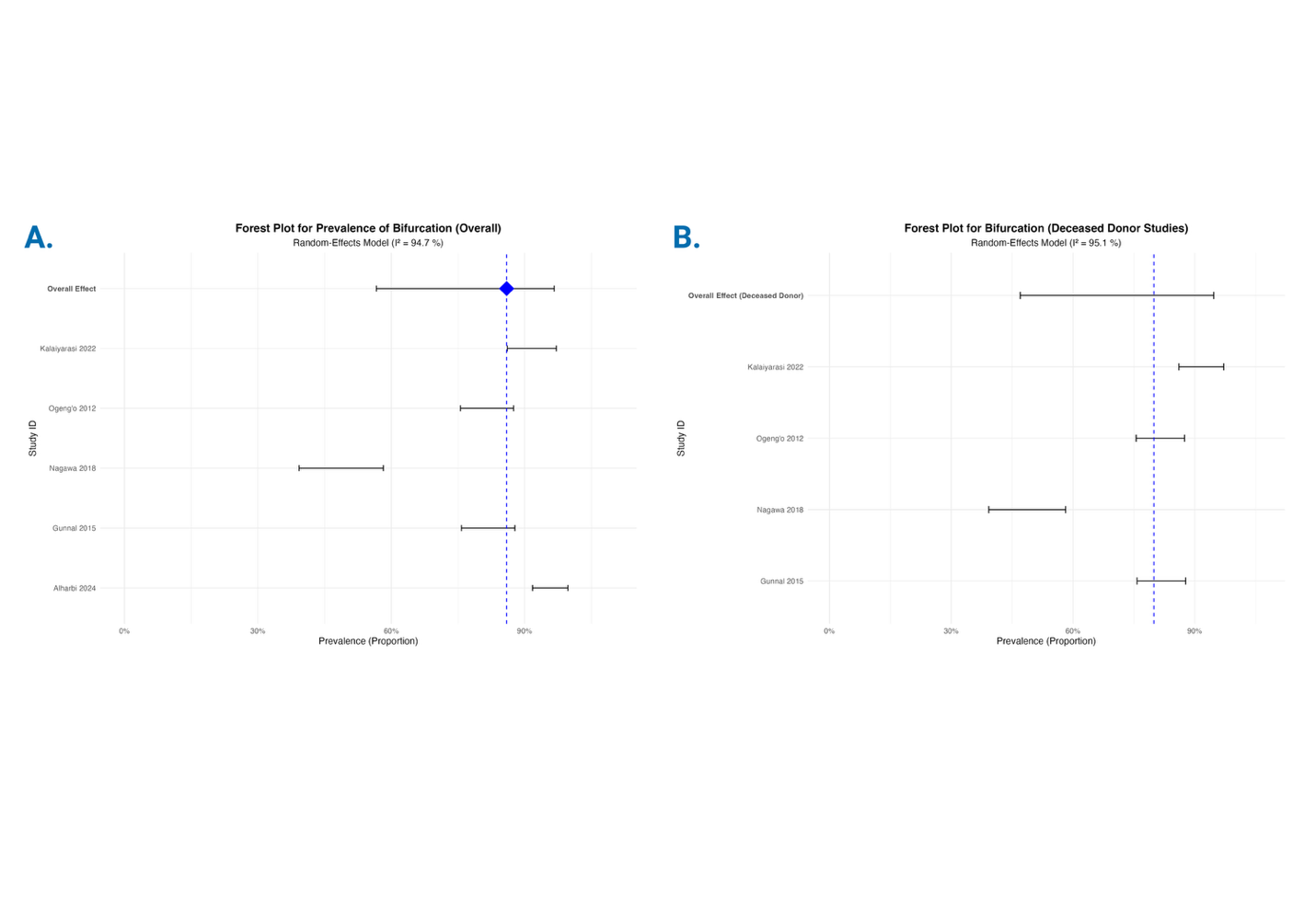


**b.**

**a.**

***Online Resource 9. Forest Plot of Bifurcation Prevalence.*** *Pooled prevalence for the classic bifurcation pattern, showing****(a)****the overall estimate (85.94%, 95% CI: 56.65–96.62%) and****(b)****the subgroup analysis for studies on deceased donors (79.94%, 95% CI: 46.99–94.71%). In the plot, horizontal lines represent the 95% confidence interval for the prevalence estimate of each study, and the diamond indicates the pooled random-effects estimate.*


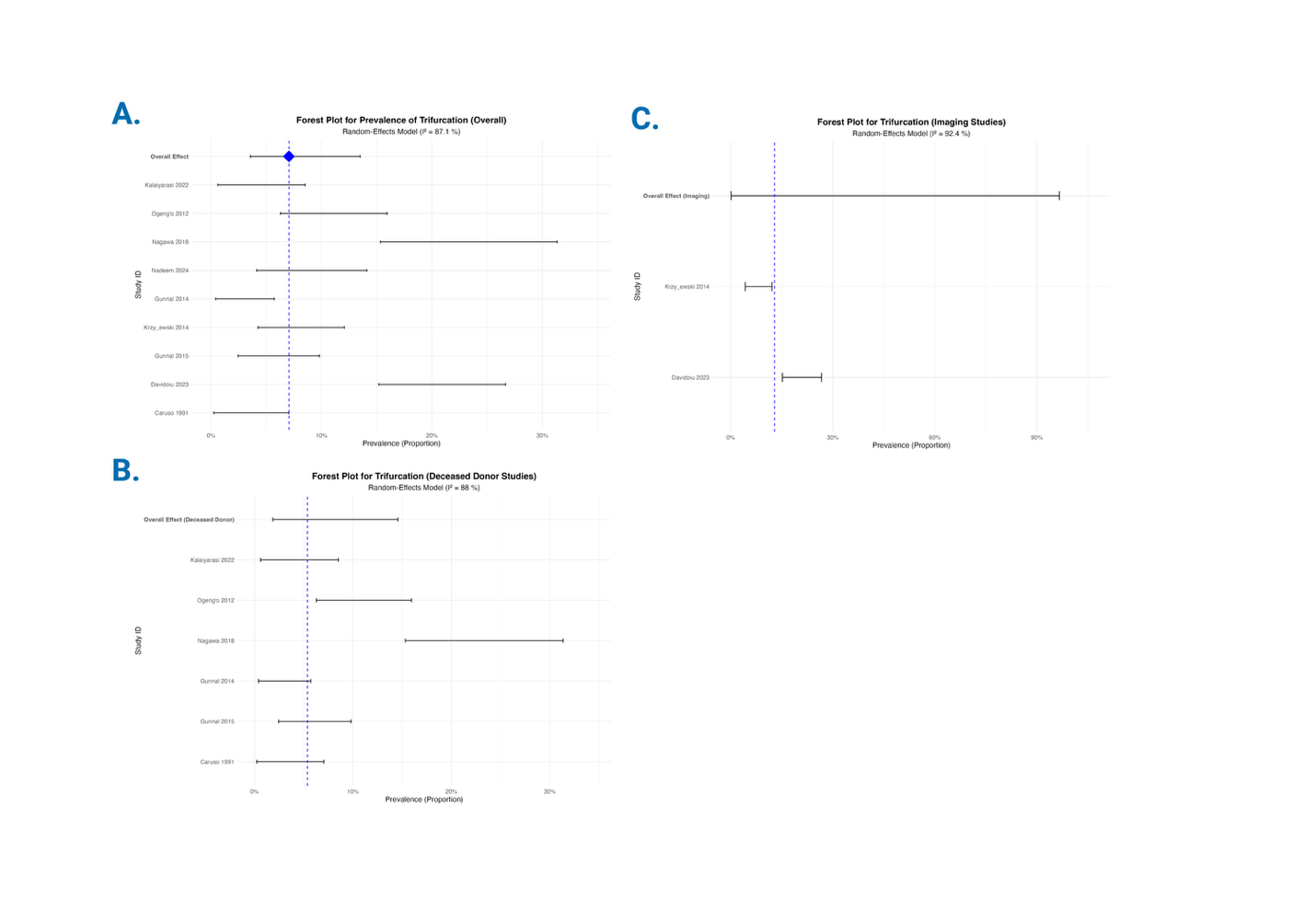


**c.**

**b.**

**a.**

***Online Resource 10. Forest Plot of Trifurcation Prevalence.*** *Pooled prevalence for trifurcation, showing****(a)****the overall estimate (7.05%, 95% CI: 3.56–13.50%), and subgroup analyses for****(b)****deceased donors (5.37%, 95% CI: 1.85–14.56%) and****(c)****imaging-based studies (12.82%, 95% CI: 0.08–96.55%). In the plot, horizontal lines represent the 95% confidence interval for the prevalence estimate of each study, and the diamond indicates the pooled random-effects estimate.*


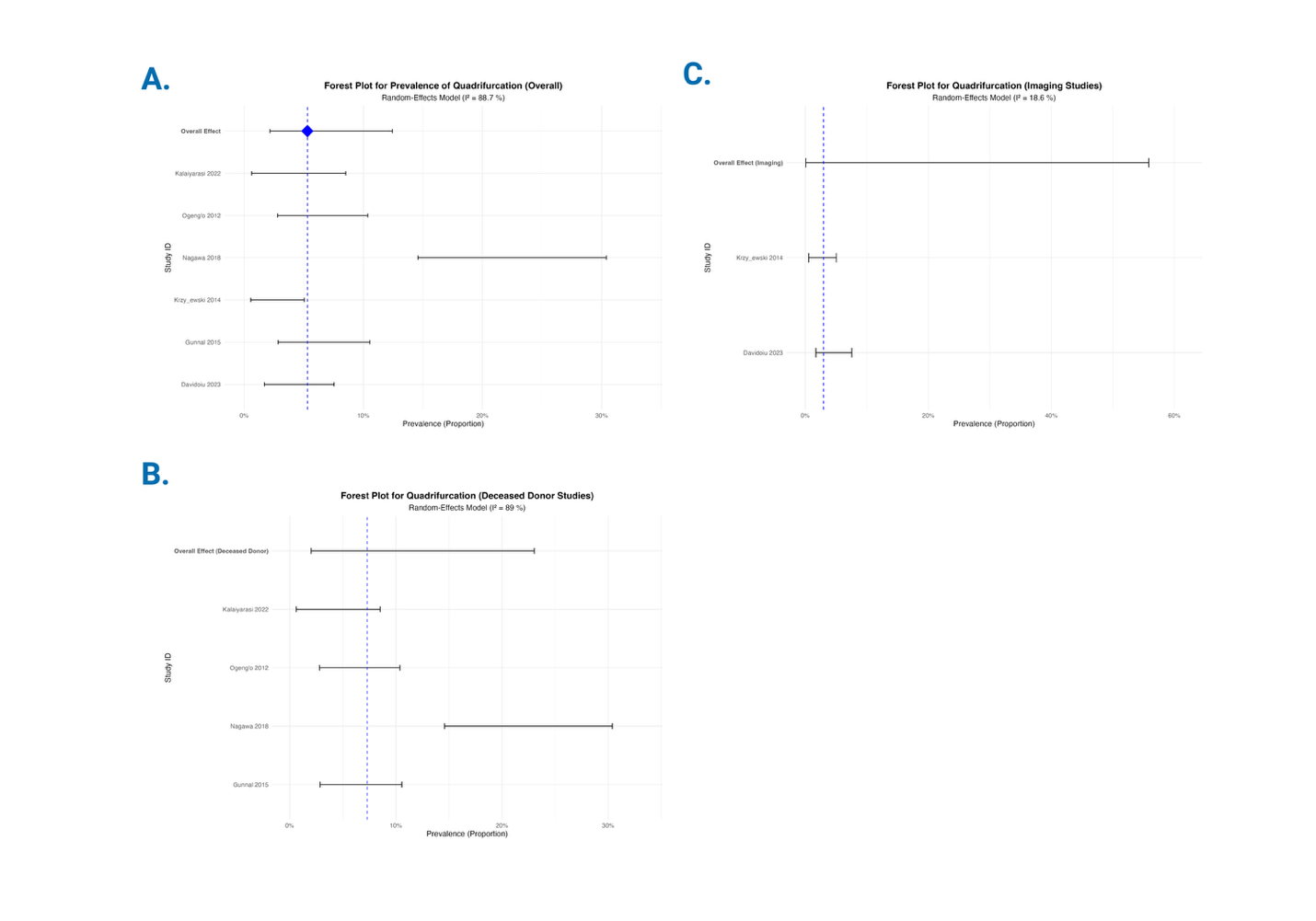


**c.**

**b.**

**a.**

***Online Resource 11. Forest Plot of Quadrifurcation Prevalence.*** *Pooled prevalence for quadrifurcation, showing****(a)****the overall estimate (5.30%, 95% CI: 2.16–12.45%), and subgroup analyses for****(b)****deceased donors (7.30%, 95% CI: 2.03–23.02%) and****(c)****imaging-based studies (2.96%, 95% CI: 0.07–55.84%). In the plot, horizontal lines represent the 95% confidence interval for the prevalence estimate of each study, and the diamond indicates the pooled random-effects estimate.*


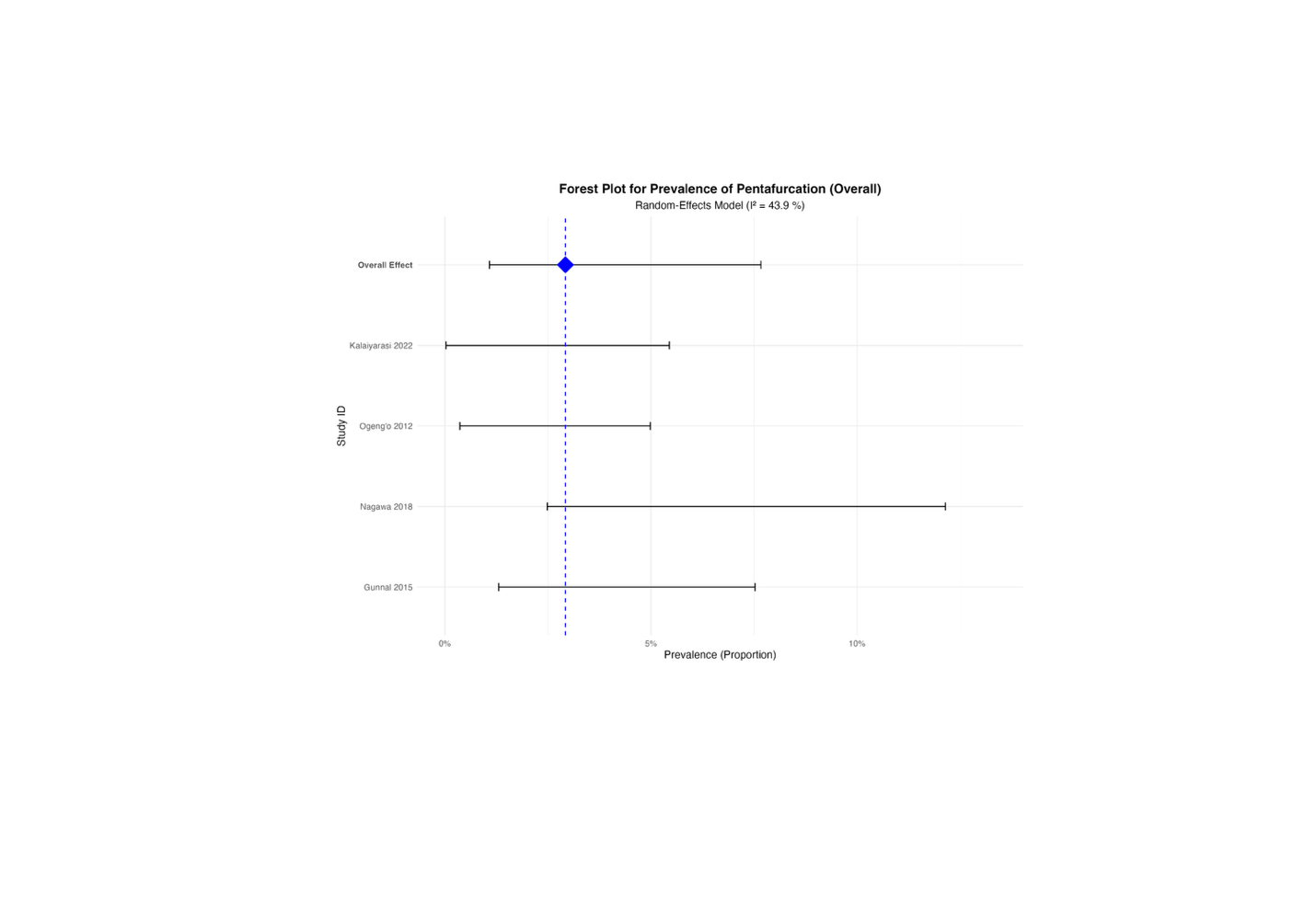


***Online Resource 12. Forest Plot of Pentafurcation Prevalence.*** *Overall pooled prevalence for pentafurcation (2.92%, 95% CI: 1.08–7.67%), derived entirely from studies on deceased donors as no data were available for other subgroups. In the plot, horizontal lines represent the 95% confidence interval for the prevalence estimate of each study, and the diamond indicates the pooled random-effects estimate.*
